# Faster Sound Stream Segmentation in Musicians than in Nonmusicians

**DOI:** 10.1371/journal.pone.0101340

**Published:** 2014-07-11

**Authors:** Clément François, Florent Jaillet, Sylvain Takerkart, Daniele Schön

**Affiliations:** 1 Cognition and Brain Plasticity Unit, Institute of Biomedicine Research of Bellvitge, Barcelona, Spain; 2 Department of Basic Psychology, University of Barcelona, Barcelona, Spain; 3 Institut de Neurosciences de la Timone, Unité Mixte de Recherche 7289, Aix-Marseille Université, Centre National de la Recherche Scientifique, Marseille, France; 4 Institut de Neurosciences des Systèmes Unité 1106, Aix-Marseille Université, Institut National de la Santé Et de la Recherche Médicale, Marseille, France; Baycrest Hospital, Canada

## Abstract

The musician's brain is considered as a good model of brain plasticity as musical training is known to modify auditory perception and related cortical organization. Here, we show that music-related modifications can also extend beyond motor and auditory processing and generalize (transfer) to speech processing. Previous studies have shown that adults and newborns can segment a continuous stream of linguistic and non-linguistic stimuli based only on probabilities of occurrence between adjacent syllables, tones or timbres. The paradigm classically used in these studies consists of a passive exposure phase followed by a testing phase. By using both behavioural and electrophysiological measures, we recently showed that adult musicians and musically trained children outperform nonmusicians in the test following brief exposure to an artificial sung language. However, the behavioural test does not allow for studying the learning process *per se* but rather the result of the learning. In the present study, we analyze the electrophysiological learning curves that are the ongoing brain dynamics recorded as the learning is taking place. While musicians show an inverted U shaped learning curve, nonmusicians show a linear learning curve. Analyses of Event-Related Potentials (ERPs) allow for a greater understanding of how and when musical training can improve speech segmentation. These results bring evidence of enhanced neural sensitivity to statistical regularities in musicians and support the hypothesis of positive transfer of training effect from music to sound stream segmentation in general.

## Introduction

Comparing musicians to nonmusicians allows studying the effects of intensive multimodal training on brain plasticity by determining the functional and structural modifications fostered by musical practice. Psychophysical studies have shown that musicians have lower perceptual thresholds than nonmusicians for frequency and temporal changes [1]–[3]. These differences might be underpinned by functional and/or structural differences in the auditory neural circuitry. It is now well established that musical practice induces functional changes as reflected by cortical and sub-cortical electrophysiological responses to auditory stimuli. Compared to nonmusicians, musicians show larger N1 and P2 amplitude (Event-Related Potentials (ERPs) generated in the auditory cortex) when listening to synthetic or instrumental sounds [4], [5]. Musicians are sensitive to sound spectral complexity (or richness): they show larger N1m to piano sounds than to pure tones, while nonmusicians are not sensitive to this contrast [6]. Additionally, it has been shown that compared to nonmusicians, musicians have larger Mismatch Negativity (MMN) elicited by deviant chords inserted in a stream of repeated standard chords [7], [8] as well as when a sound is omitted in the stream [9]. These differences point to the greater efficiency of musicians' auditory system in processing sound features. While these musician advantages were primarily observed in music-related tasks, some studies have shown that this advantage could generalize to speech sound processing. Indeed, musicians show more robust encoding of speech sounds in the brainstem [10]–[14]. Both adult and children musicians better detect fine contour modifications in the prosody of an utterance than matched controls [15], [16]. Recent studies have also shown that musical practice improves the sensitivity to durational changes in both speech sounds and utterances [17], [18]. These findings are supported by other studies showing positive correlations between musical and linguistic aptitudes in children and adults [19]–[22]. The focus of the present work is the time course of speech segmentation, the ability to extract words from continuous speech. Natural speech contains several acoustic cues such as pauses or lexical stresses that are useful for the detection of word boundaries [23]. Nonetheless, there is evidence showing that an artificial speech stream without any consistent acoustic cue can be segmented in an implicit manner based on the statistical structure of the language [23], [24]. In general, “syllables that are part of the same word tend to follow one another predictably, whereas syllables that span word boundaries do not” [25]. The role of conditional probabilities (the probability of syllable X given syllable Y) in segmenting a speech stream of nonsense pseudowords has been shown in neonates, infants and adults [23], [24], [26]–[29]. Throughout this series of studies, the authors showed that listening to an artificial language without acoustic cues at word boundaries yields correct word recognition in a subsequent behavioural test. Participants discriminated pseudo-words that were part of the language from similar pseudo-words that were not part of the language. Importantly, this learning paradigm has been replicated using sung syllables [30], non-linguistic stimuli such as sounds with different pitches [31], [32] or timbres [33] as well as with nonsense sounds [34] and morse-code like sounds [35], thus pointing to a domain general rather than a language specific mechanism.

Recently, we analyzed ERPs recorded during the behavioural test that immediately followed the exposure phase. We found a late fronto-central negative component that was larger for unfamiliar than for familiar pseudo-words. We interpreted this familiarity effect as a greater difficulty in accessing unfamiliar pseudo-word representations [36]. In a further experiment [37] we compared a group of adult musicians to a group of nonmusicians. While musicians barely outperformed nonmusicians at the behavioural level, electrophysiological measures revealed a larger familiarity effect over fronto-central regions in musicians than in nonmusicians. These findings have been recently replicated in a longitudinal study with children who followed a music-training program during two school years [38]. However, data collected during the behavioural test are smeared by decisional, memory and rehearsal processes and thus, compared to data collected during the exposure phase behavioural data reflect more the result of the learning than the learning process “per se”. Previous ERP studies have revealed that, compared to high frequency words, low frequency words elicit a larger negativity peaking around 400 ms [39], [40]. The N400 amplitude is sensitive to the ease of retrieving long-term word memory traces and this ERP component has been classically interpreted as an index of lexical semantic processing [41]. Interestingly, more recent studies focusing on the on-line speech segmentation learning process reported N100 and/or P200 and/or N400 amplitude modulations as a function of exposure to the stream [42]–[44]. The EEG data showed different patterns of ERP amplitude modulations (the electrophysiological learning curves) as a function of the level of performance in the subsequent behavioural test. For instance, participants with good behavioural performance (good learners) showed an inverted U-shaped N400 learning curve: the N400 amplitude increased during the first minute of the exposure phase to reach a plateau during two minutes and finally decreased in amplitude at the end of the exposure. Additionally, middle learners presented a more linear N400 learning curve whereas low learners did not show N400 modulations [42], [45]. These results are important for refining models of language learning as they link the electrophysiological patterns of ERP modulations occurring during the exposure phase and the word recognition during the test. Moreover, according to the time-dependent model of learning, the brain areas involved in the learning of a specific skill should show increasing activation during the learning period and decreasing activation when the learning is achieved [46]. Thus, these results also provided accumulating electrophysiological evidences of the time-dependent model of learning applied to language learning.

In this study, we report the electrophysiological learning curves derived from EEG data collected during the exposure phase of a stream of artificial sung syllables. We used a sung stream to allow testing for the recognition of both linguistic and musical structures contained in the sung stream. Adult participants listened to an artificial language of sung pseudo-words and were subsequently tested with a two-alternative forced-choice task on pairs of pseudo-words and melodies (familiar vs unfamiliar, data acquired during the test have been previously described in [37]). The aim of this study was to test whether musical expertise can modify the learning process by comparing the electrophysiological learning curves of 2 groups, with or without formal musical training. Based on the time-dependent hypothesis, we expected the electrophysiological learning curves to be different in the two groups with musicians showing an early increase in N400 amplitude (supposed to indicate that a string of phonemes has been chunked) that should be followed by a decrease (supposed to indicate that a string of phonemes has been recognized) while nonmusicians showing a linear increase in N400 amplitude.

## Methods

### Ethic Statement

Written informed consent was obtained from all participants, and the data were analyzed anonymously. This study was approved by the CNRS - Mediterranean Institute for Cognitive Neuroscience and was conducted in accordance with national norms and guidelines for the protection of human subjects.

### Participants

Two groups participated in this experiment. Thirteen professional musicians (mean age 27, range 21–36, 13 right-handed, 10 males, no known neurological problems, more than 12 years of formal musical learning and from 3 to 7 hours of daily practice, 5 of them reported absolute pitch) and 13 nonmusicians (mean age 25, range 22–36, 13 right-handed, 11 males, self-reported normal hearing, no known neurological problems, no more than 2 years of formal musical training, no instrument practice during childhood). The musician participants were, at the time of the study, enrolled either in the CFMI (Centre de Formation des Musiciens Intervenants), which discerns a French diploma to teach music at primary school, or were enrolled in the CEFEDEM (Centre de Formation des Enseignants de la Musique), which discerns a French diploma to teach music at high-school and conservatory. Because of this specific training, all musician participants played at least 2 different instruments and were also proficient in singing. The two groups of participants were matched on age, sex and had similar socio-economic status. All participants were French native speakers and listened to 5.5 minutes of a continuous speech stream resulting from the concatenation of five three-syllable nonsense pseudo-words (hereafter words) that were repeated 100 times in a pseudo-random order. All participants were paid 20 Euros.

### Material

The artificial language consisted of four consonants and three vowels, which were combined into a set of 11 syllables with an average length of 230 ms (sd  = 16 ms). Each of the 11 syllables was sung with a distinct tone (C3, D3, F3, G3, A3, B3, C4, Db4, D4, E4, and F4). These 11 syllables were then combined to give rise to five trisyllabic sung pseudo-words (gimysy, mimosi, pogysi, pymiso, sipygy). Therefore each pseudo-word of the language was always sung on the same melodic contour (gimysy C3 D3 F3, mimosi E4 Db4 G3, pymiso B3 E4 F4, pogysi D4 C4 G3, sipygy G3 B3 C4). The mean pitch interval within pseudo-words was not significantly different from the mean interval between pseudo-words (p = .4). No pitch-contour changes occurred within the pseudo-words (3 pseudo-words contained a rising pitch-contour while 2 contained a falling pitch-contour). Moreover, pitch-contour changes could not be used to segment the stream as this cue was not consistent: only half of the word boundaries were marked by pitch-contour changes. Because some of the syllables appeared in multiple words, transitional probabilities within words ranged from 0.5 to 1.0. Transitional probabilities across word boundaries ranged from 0.1 to 0.5. The language stream was built by a random concatenation of the five pseudo-words (only constraint: no repetition of the same item twice in a row) and synthesized using Mbrola (http://tcts.fpms.ac.be/synthesis/mbrola.html). No acoustic cues were inserted at word boundaries. Each word was repeated 94 times in the stream leading to a 5.5 minute continuous speech stream. In the linguistic test, test items consisted of the five pseudowords used in the exposure phase and five foils synthetized with a flat contour (spoken version). In the musical test, test items consisted of piano melodies with the same pitches defining the melodic contour of the pseudowords and their corresponding foils. The foils items contained either the last syllable (or pitch) of a pseudoword plus the first syllable (or pitch) pair of another pseudoword or the last syllable (or pitch) pair of a pseudoword plus the first syllable (or pitch) of another pseudoword.

### Design and Procedure

**Figure 1:**
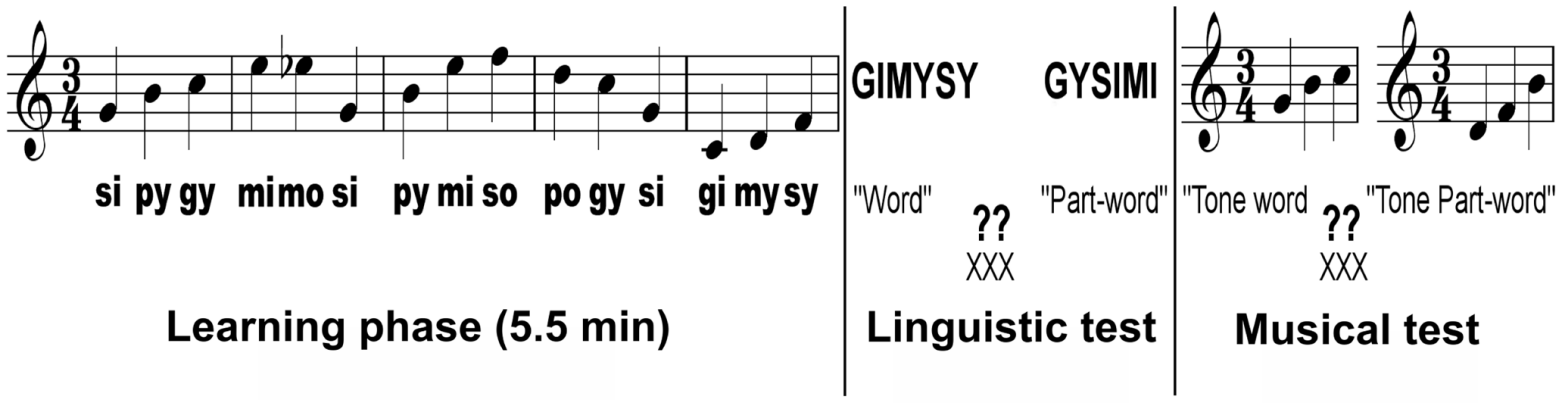
Illustration of the experimental design used in the present experiment. Stimuli were presented auditorily via loudspeakers. The learning phase lasted 5.5 minutes and the order of the tests was counter balanced across participants.

Before the learning phase, participants were told they would have to carefully listen to a continuous stream of sung syllables for several minutes because they would be quizzed after this exposure phase. No explicit instruction on word learning was given and we did our best to keep the entire procedure implicit. During the behavioural test, the participants had to choose, by pressing one of two response buttons, which of two strings (first or second tri-syllabic pseudo-word) most closely resembled what they just heard in the stream. Test items had a flat contour (“spoken” version) in the linguistic test while they were played with a piano sound in the musical test (Figure 1). In each test trial, one item was a “pseudo-word” (linguistic test) or “melody” (musical test) from the artificial language (hereafter familiar word/melody) while the other item was a foil. Stimuli were presented via loudspeakers. Linguistic and musical tests lasted 5 minutes each and their order was counter-balanced across participants.

### EEG data acquisition

The participants were comfortably seated in a Faraday booth. EEG data were continuously recorded from 32 active Ag-Cl electrodes (Biosemi ActiveTwo system, Amsterdam University) located at standard left and right hemisphere positions over frontal, central, parietal, occipital, and temporal areas (International 10/20 system sites: Fz, Cz, Pz, Oz, Fp1, Fp2, AF3, AF4, F3, F4, C3, C4, P3, P4, P7, P8, Po3, Po4, O1, O2, F7, F8, T7, T8, Fc5, Fc1, Fc2, Fc6, Cp5, Cp1, Cp2, and Cp6). The electro-oculogram (EOG) was recorded from Flat-type active electrodes placed 1 cm to the left and right of the external canthi, and from an electrode beneath the right eye. The band-pass was of 0–102.4 Hz and sampling rate 512 Hz.

### ERP analyses

Six participants were discarded due to major artifacts, thus yielding to two groups of 10 participants each. Major artifacts were due to excessive environmental noise such as constructions taking place at the floor below the EEG room (4) and low drifts possibly due to sweating (2). The EEG data, acquired continuously during the exposure phase, were then re-referenced offline to the algebraic average of the left and right mastoids. Signal containing ocular artifacts was corrected using ICA decomposition by removing the component containing the blink [47]. The full EEG recording was first divided into 4 non-overlapping consecutive time bins of 1′20″ duration. The EEG was then segmented in epochs of 750 ms starting 50 ms prior to pseudo-words onsets. A −50 to 0 ms baseline zero-mean normalization was applied using Brain Vision Analyzer software (Brain Products, Munich). Artifact rejection was then carried out on epoched data for each subject using a statistical threshold (excluding epochs with an absolute value exceeding the mean of all trials +2.5 σ). Based on the literature and on visual inspection of the ERPs, statistical analyses of the N1 and P2 components were performed on the mean amplitude computed in the 100–170 and 200–300 ms latency bands respectively. Statistical analyses of the N400 component were performed on the mean amplitude in the 350–550 ms latency band. Repeated Measure Analysis of Variance (ANOVAs) was used for statistical assessment with Expertise (musicians *vs*. nonmusicians) as between-subjects factor and time bin as within-subject factor (4 consecutive non-overlapping time windows of 1'20'', 114 trials each). Topographical distribution of the effects was modeled by 2 additional factors (Hemisphere, left and right and Antero-posterior, frontal, central, and parietal) defined as follows: left (AF3, F3, F7) and right (AF4, F4, F8) frontal, left (Fc1, C3, Fc5) and right (Fc2, C4, Fc6) central, and left (Po3, P3, P7) and right (Po4, P4, P8) parietal. All P values reported below reflect the difference between the first time bin and each subsequent bin. All P values were adjusted using the Greenhouse-Geisser correction for nonsphericity, when appropriate, and Fisher tests were used in post-hoc.

## Results

### Behavioural data

**Figure 2:**
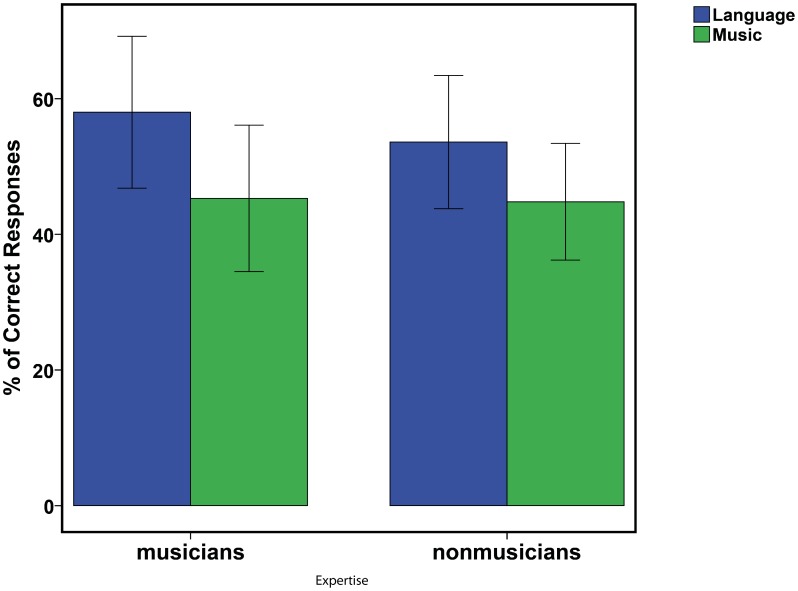
Percentage of correct responses. Group performance in the linguistic (green) and musical tests (blue) for musicians (left) and nonmusicians (right). The error bars represents +/− Standard Error.

Results of a two-way Repeated-measure analysis of variance (ANOVA) [Expertise (as between factor with 2 levels) and Dimension (Linguistic and Musical tests, as within factor with 2 levels)] showed a main effect of dimension [F (1, 18) = 14.82; p<.001]: the linguistic dimension was learned better than the musical one for both groups (Figure 2). The main effect of Expertise and the Expertise by Dimension interaction were not significant (F's <1). Comparison of performance in the linguistic test with chance level (here 50%) showed that musicians learned the pseudo-words contained in the stream but not nonmusicians (58% and 54% of correct responses, T(10) = 6.5; Z = 2.14; p = .03 and T(10) = 18.5; Z = 0.91; p = .35 respectively, Wilcoxon tests). However, direct comparison of the performance of two groups did not reach significance. In the musical test, the level of performance in both groups was below chance level but this difference was not statistically significant (musicians: 46% of correct responses; nonmusicians: 44%, both p's >.1), showing that participants did not learn the musical dimension contained in the stream.

### ERP data

#### N1 analyses

The main effect of time-bin was significant [F (3,54) = 6.14; p = .003]. The N1 amplitude was maximal during the first time-bin (−0.32 µV) and post-hoc analyses showed that, compared to the first time-bin, N1 amplitude significantly decreased throughout stream exposure (2^nd^ time-bin: 0.37 µV; p = .002; 3^rd^: 0.39 µV; p = .001 and 4^th^: 0.51 µV; p<.001). While the main effect of Expertise and of Hemisphere were not significant (F = .32 and 3.78 respectively), the main effect of Antero-posterior gradient was significant [F (2, 36) = 10.28; p = .001] with significantly larger N1 amplitude over parietal (−.04 µV) than over frontal and central regions (0.34 and 0.41 µV respectively; both p's<.001). The time bin by Antero-posterior gradient as well as the time bin by Expertise interactions were not significant (F = 1.30 and .53 respectively).

#### P2 analyses

No modulation of the P2 component as a function of exposure was found in the analyses (main effect of time bin: F<1). The main effect of Expertise was not significant (F<1).

#### N400 analyses

**Figure 3:**
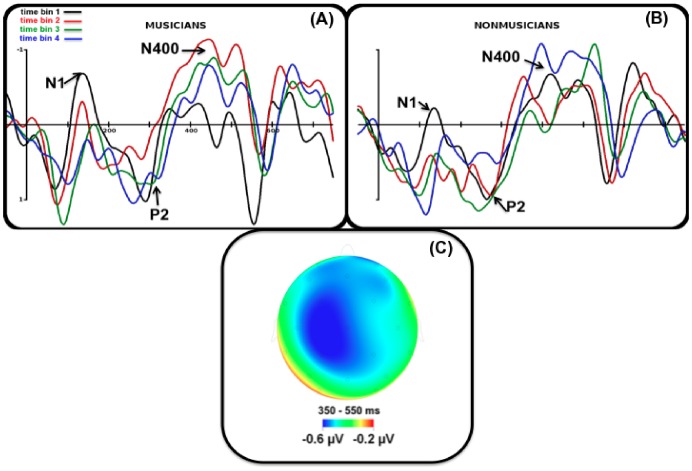
Grand average (Fronto-Central region) across musicians (A, left) and nonmusicians (B, right) recorded during each time bin of the exposure phase. (black = 1st time bin, red = 2nd time bin, green  = 3rd time bin, blue  = 4th time bin). (C) Map showing the distribution of the N400 component (350–550 ms latency band, averaged across time bins and groups).

**Figure 4:**
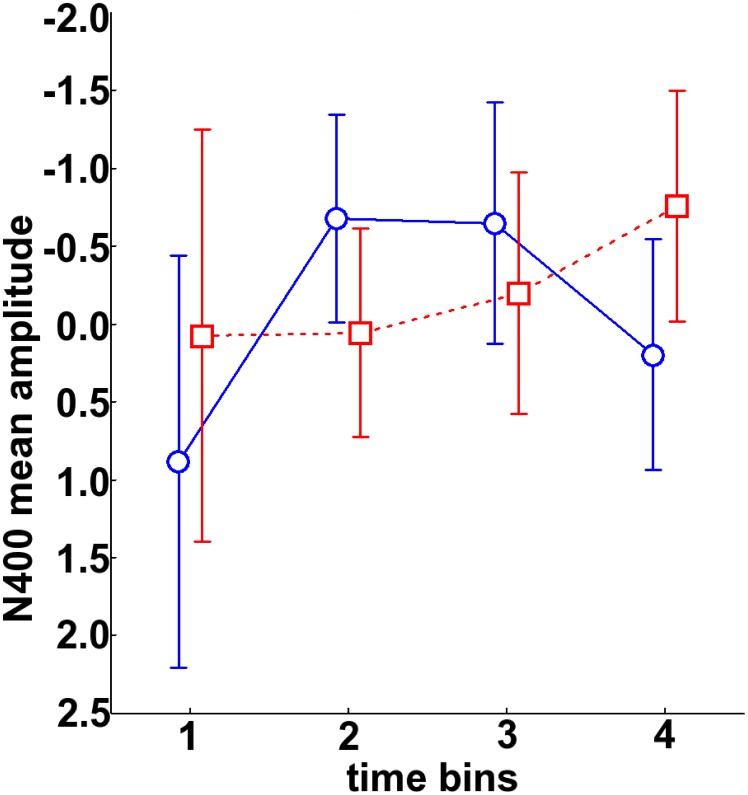
N400 mean amplitude (350–550 ms) averaged across 6 fronto-central electrodes in both groups of participants (musicians in blue, nonmusicians in red) and in the four time bins (1'20'') from the exposure phase. Negativity is up. Error bars refer to confidence intervals computed as described in [48] and take into account inter-subject variability, separately for each group.

N400 mean amplitude modulations were different in the two groups (Expertise by time bin interaction [F (3, 54) = 3.65; p = .02], Figure 3A, 3B). Musicians showed an inverted U-shaped N400 learning curve: compared to the first time bin, the N400 mean amplitude significantly increased in the 2^nd^ (−0.41 µV; p = .03) and 3^rd^ time bin (−0.37 µV; p = .04) and then decreased during the 4^th^ time bin (0.10 µV; p = .88). By contrast, nonmusicians showed a linear N400 learning curve: the N400 mean amplitude increased through exposure reaching a marginally significant increase during the 4th time bin (first time bin: −0.16 µV; 2^nd^ time bin: −0.04 µV, p = .65; 3^rd^ time bin: −0.21 µV, p = .85 and 4^th^ time bin: −0.68 µV, p = .06). Based on visual inspection of the scalp distribution of the N400 component across time bins and groups and because the Expertise by Time bin by Anteroposterior interaction was significant ([F (6, 108) = 4.33; p = .01]), an additional analysis was conducted on 6 fronto-central electrodes (i.e. where N400 amplitude was maximum, Figure 3C). For musicians, compared to the first time bin, the N400 significantly increased in the 2^nd^ (−0.68 µV; p = .02) and 3^rd^ (−0.64 µV; p = .03) time bins and decreased during the 4^th^ time bin (0.19 µV; p = .80). For nonmusicians, the N400 increased linearly through exposure and reached maximum amplitude during the 4^th^ time bin (−0.75 µV; p = .07; Expertise by time bin interaction F (3, 54) = 3.54; p = .04, Figure 4). In order to test whether electrophysiological learning curves were linear or not we performed a linear regression analysis including N400 amplitude measures (on the 6 fronto-central electrodes) as dependent variable and time bin as predictive factor for musicians and nonmusicians separately. Results showed that this regression was significant for nonmusicians [F (1, 38) = 4.87, p = .03] but not for musicians [F (1, 38) = 0.90; p = .35]. By contrast polynomial regression using a quadratic function [f (x) = ax^2^+bx+c] showed that exponential parameter estimates were only significant for musicians [F (1, 38) = 7.4; p = 0.009] and not for nonmusicians [F (1, 38) = 0.93; p = .33].

### Brain-Behaviour Correlation

**Figure 5:**
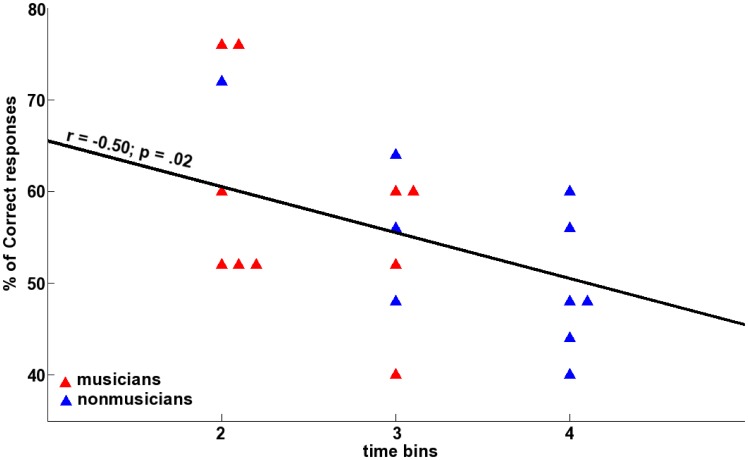
Scatter plot of accuracy in the behavioural test versus the time bin showing the maximum N400 amplitude. Regression index and the p value are provided on the plot. Musicians are represented in blue and nonmusicians in red.

We found a significant correlation between accuracy in the linguistic test and the time bin during which the N400 mean amplitude was maximum (r  = −0.50; p = .02; Spearman correlation; Figure 5). The level of performance was higher in participants showing maximum N400 amplitude early on during exposure (i.e. 2^nd^ time bin).

We run an additional analysis using a stepwise regression with the performance in the linguistic task as dependent variable and the maximum amplitude of the N400, the increase in N400 (compared to the first time bin) and the time bin showing the maximum N400 amplitude as predictive variables. This analysis revealed that while the maximum amplitude of the N400 and the increase in N400 were not good predictors of the level of performance in the linguistic test (N400 amplitude: β = 0.24, t (16) = 1.18, p = .25; N400 increase: β = 0.13, t (16) = 0.67, p = .51), the time bin showing the maximum N400 amplitude was a significantly good predictor (Time Bin of N400 max: β = 0.49, t (16) = 2.34, p = .03). This strongly suggests that the dynamics of the N400 amplitude played an important role here.

## Discussion

The goal of the present study was to test whether musical expertise can modify the on-line neural correlates of speech segmentation. Both musicians and nonmusicians showed a progressively emerging fronto-central negative component in the 350–550 ms latency band. Nonetheless, while musicians showed an inverted U-shaped N400 curve, nonmusicians showed a rather linear N400 curve (see Figure 4). Interestingly, the level of performance in the linguistic test could be predicted as a function of the time bin having the maximum N400 amplitude; participants for whom the N400 reached its maximum in an early time bin had a higher level of performance that those where the N400 amplitude reached its maximum later (see Figure 5).

The behavioural results confirm our previous study with adults and children [37], [38], [49] as well as other recent evidence showing that musicians outperform nonmusicians in implicit segmentation tasks [35], [50], [51], possibly due to a greater sensitivity to the statistical properties of the auditory input stream in experts than in non experts [52]. We found no evidence of learning in either group in the music condition. This is probably partly due to a lack of musical significance in the stream and most importantly to a greater interference in the musical test due to the presence of foils (spanning word boundaries) that are highly competing with the melodies of the language due to the relative nature of pitch sequences (intervals). This lack of learning of the musical dimension in both groups is important because it supports the notion that the learning effect in musicians in the language dimension was not driven by musical characteristics of the words.

Of great interest here is the fact that the participants who were most accurate on the linguistic test were those showing maximum N400 amplitude early in the exposure phase. Moreover, neither the maximum amplitude of the N400 nor the increase in N400 amplitude predicted the level of performance in this test. These results are important for two reasons. First, they show that musicians and nonmusicians not only have different segmentation abilities, but that these skills rely on different neural dynamics as estimated from EEG during the exposure phase. Second, N400 modulations are a powerful predictor of the success in the following test. This means that a completely implicit and non-interfering measure such as the dynamics of the N400 during passive exposure can be a valuable indicator of speech segmentation competences. This finding may have in turn strong implications in fundamental and clinical research when working for instance with babies, young children or pathologic populations (e.g. patients with executive functions or speech disorders). Finally, the different patterns of ERP modulations found in these 2 groups extend our knowledge on general theories of learning such as the time-dependent hypothesis of learning.

### Faster word extraction in Musicians than in nonmusicians

Modulations of the amplitude of early ERP components (N1 and/or P2) during exposure have been previously described in nonmusicians using similar paradigms [44], [53]. Recently, an effect of musical practice was found on the P50 component using a stream of tones [54]. In the present study, while during the first minute of exposure (first time bin), musicians seem to show larger N1 than nonmusicians, this difference did not reach significance. This discrepancy with previous research may be due to the acoustic features of the stimuli used in our study; the set of consonants we used had heterogeneous attack times probably resulting in larger ERP latency variability compared to studies using piano tones for instance. Future experiments will be needed to confirm the involvement of these early ERP components in the segmentation process and their interactions with musical expertise.

Nonetheless, despite a lack of significance on the early ERP components, the dynamic patterns of N400 modulations along the exposure phase clearly differentiated the two groups before the behavioural test: musicians showed an inverted U-shaped N400 amplitude curve while a linear N400 amplitude curve was observed in nonmusicians. A previous study using both EEG source reconstruction and fMRI with a similar artificial language learning (ALL) paradigm has described the middle temporal gyrus as a possible generator of this fronto-central component [43]. The fact that no learning related modulations were found on auditory ERP components whereas we found modulations on the N400 component suggests that the difference between the 2 groups goes beyond the auditory cortices possibly at the level of the superior temporal plane [55] and middle temporal gyrus [43].

Musicians showed a significant increase in N400 amplitude as soon as the second time bin of the exposure phase (i.e. between 1'20'' and 2'40''). Previous studies using similar artificial language learning paradigms with speech and tone streams have reported a similar steep increase in N400 amplitude after 2 minutes of exposure in the group of good learners only [43]. This N400 increase has been interpreted as reflecting the building of proto-lexical representations. While at the beginning the parsing unit is possibly the syllable, due to the statistical properties of the material the three syllables comprising a given word are little by little perceived as a unique pattern: a new word candidate. Thus, a faster N400 increase in musicians points to a faster ability to take advantage of the statistical structure of the stream to segment the words. Interestingly, the superior temporal plane seems to be sensitive to the statistical regularities of the input [55] and metabolic activity within this region is positively related to participants' ability to recognize words during the behavioural test of a similar artificial language learning experiment [56]. Importantly, at the structural level, musicians show larger planum temporale than nonmusicians [57], [58]. Thus, the anatomo-functional reorganization induced by musical practice within this region may well be at the origin of musicians' superiority in speech segmentation. Additionally because the speech stream used was sung, it might be that musicians were more sensitive to the pitch patterns contained in the speech stream than nonmusicians. However, as previously mentioned, the lack of learning in the musical dimension supports the notion that the learning effect reported in musicians in the language dimension was not driven by musical characteristics of the words. Rather musicians may take advantage of their rhythmic skills that may allow them to orient attention at the most salient time points of the stream (word boundaries). In other words, as long as attention remains "entrained" at the syllable level, words are not segmented. As soon as attention is oriented at longer time windows (here three syllables), words may start to pop out of the stream.

The steep increase in N400 amplitude was immediately followed by a 2-minute asymptote that could reflect the saturation of the network. This N400 plateau could reflect the consolidation of word memory traces within a fronto-temporal network allowing for later word recognition. One may make the hypothesis that increasing the duration of the exposure phase for nonmusicians would result in a similar but delayed asymptote. In other words the neural mechanisms of this type of learning are probably not fundamentally different in musicians and nonmusicians. Differences would simply be quantitative, with musicians having a faster segmentation than nonmusicians; comparing musicians to non-musicians who were equally good language learners one would expect the learning curves to be similar. Interestingly, this is the case for the one nonmusician having a good behavioural performance (72% correct) who also shows a peak of N400 amplitude at the second time bin. This gives again the impression that the U-shape curve does predict learning to some extent.

An alternative explanation of this asymptote could rely on the implication of the working memory system and in particular its articulatory rehearsal subcomponent that has been shown to play an important role in speech segmentation and word learning [43], [56]. Indeed, disrupting the rehearsal mechanism with an articulatory suppression procedure along the exposure phase leads to unsuccessful word segmentation [59]. Interestingly, a recent study has revealed that musicians have better functioning and faster updating of working memory than nonmusicians [60]. In the same vein, it has been shown that compared to nonmusicians, musicians can hold more information and for longer periods in their auditory memory [61]. Thus, musicians may have been relying more on an articulatory rehearsal mechanism than nonmusicians leading to better word segmentation. Because there is now evidence of greater working memory in musicians [60], [61], future research will need to bridge working memory and segmentation abilities and the extent to which inter-individual differences in working memory may subsequently drive differences in segmentation abilities.

Finally, the last 2 minutes of the exposure phase showed a decrease in N400 amplitude in musicians but not in nonmusicians. A similar decrease has been reported in two previous studies on ALL and on tone stream segmentation [43], [45]. Additionally, when a word is known, its familiarity and repetition will typically engender a reduction in N400 amplitude [39], [40], [62]. In the case of ALL experiments, a decrease in N400 amplitude has also been interpreted as reflecting a phonemic template pattern matching/recognition process probably involving the Inferior Frontal Gyrus/PreMotor Cortex complex (IFG/PMC) [43], [63]. Interestingly, this area is also involved in harmonic music perception [64], [65] and has an increased gray matter density and volume in musicians compared to nonmusicians [66].

Finally, musical practice has been shown to increase both structural and functional connectivity within the speech-processing network in patients recovering from stroke [67] and in children [68]. Both adult musicians and 8-year old children who followed 2 years of musical training show a more developed arcuate fasciculus than nonmusicians [68]–[70]. This fiber bundle is crucial in the mapping of speech sounds to articulatory gestures by connecting the posterior part of the Superior Temporal Gyrus to the IFG/PMC [71], [72]. Lesions of the arcuate fasciculus induce impairment not only of phonological and word repetition but also in verbal short-term memory [73]–[75]. Interestingly, a recently published study has revealed that the arcuate fasciculus is crucial in mediating word learning [76]. Thus, increased connectivity between auditory and motor regions might lead to better segmentation skills.

To conclude, the present results bring new evidence showing that musicians are not only better but also faster at segmenting an artificial language compared to nonmusicians. The modulation of the purported neural correlates of learning were evident earlier in the exposure phase in musicians than in nonmusicians suggesting that word segmentation is achieved more quickly during the exposure phase. The different patterns of ERP modulations during exposure as well as the significant correlation with behavior in a following test provide additional validity to the time-dependent hypothesis stating that an increasing activation of the network sustaining a specific learning process should be limited to the initial learning periods and should not be visible after the learning is accomplished [46].
